# Comparing Interface Conditions for a 3D–0D Multiscale Interface Coupling With Applications in Tissue Perfusion

**DOI:** 10.1002/cnm.70017

**Published:** 2025-02-14

**Authors:** L. Bociu, M. Broussard, G. Guidoboni, D. Prada, S. Strikwerda

**Affiliations:** ^1^ Department of Mathematics NC State University Raleigh North Carolina USA; ^2^ Maine College of Engineering and Computing University of Maine Orono Maine USA; ^3^ Istituto di Matematica Applicata e Tecnologie Informatiche “Enrico Magenes” Consiglio Nazionale delle Ricerche Pavia Italy; ^4^ Department of Mathematics University of Pennsylvania Philadelphia Pennsylvania USA

**Keywords:** lumped hydraulic circuit, multiscale interface coupling, operator splitting, poroelasticity

## Abstract

Many pathologies are related to hemodynamic changes occurring at the microvascular level, where small vessels pierce the tissue, perfusing it with blood. Since there is a large number of vessels of small caliber, it is impractical to model the fluid flow through each one of them separately, as it is done in the case of large arteries using, for example, the Navier–Stokes equations. As an alternative, tissue perfusion is modeled here via three‐dimensional (3D) partial differential equations (PDEs) for fluid flow through deformable porous media, where blood vessels are modeled as pores within a deformable solid representing the tissue. Since it is known that the local perfusion is related to the systemic features of surrounding blood circulation, we couple the PDE system with a zero‐dimensional (0D) lumped circuit model, obtained by the analogy between fluid flows in hydraulic networks and current flowing in electrical circuits. An important feature in this multiscale 3D–0D coupling is the specification of interface conditions between the 3D and the 0D parts of the system. In this article, we focus on two types of interface conditions driven by physical considerations, and compare the behavior of the solutions for the two different scenarios.

## Graphical abstract

Test 7.2: streamlines of the fluid velocity in the region {x = (x, y, z)∈ Ω | 0.49 ≤ x ≤ 0.5, z ≤ 0} at time t = 10 s, time step Δt = 0.02 s. Color map represents magnitude of the velocity (units = m/s).

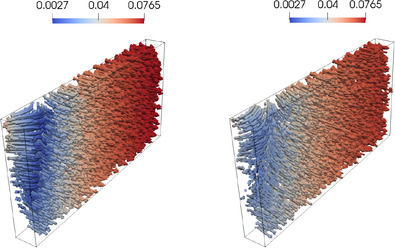

## 1 Introduction

We consider a multiscale model based on the coupling of partial and ordinary differential equations (PDEs and ODEs) with applications in tissue perfusion (i.e., delivery of blood to a capillary bed in tissue). The main idea is the following: we have a local region of interest (like the perfusion of a tissue, or organ, etc.), for which we provide a detailed, accurate three‐dimensional (3D) description, while at the same time we account for the global features of the problem (like the surrounding blood circulation) using a reduced zero‐dimensional (0D) representation of a hydraulic network. This “low cost” model is derived using the analogy with electrical circuits, and it predicts the evolution in time of average quantities like pressure and flow rate.

This multiscale strategy has been successfully used for studying physiological flows (like blood flows in arteries or air flow in the bronchial tree). In these scenarios, Stokes or Navier–Stokes equations described the blood flow in a sensible region (e.g., a stented artery, or coronary bypass), while systemic 0D lumped circuit models approximated the remainder of the circulation [1, 2, 3, 4, 5, 6, 7, 8, 9, 10]. In comparison, in this paper we focus on biological models where the perfusion of a specific tissue or organ (which is the local phenomenon) is studied in correlation to the global features of the surrounding blood circulation [11, 12, 13, 14, 15, 16].

The motivation for our investigation comes from the fact that many pathologies are related to the hemodynamic changes that occur at the microvascular level, where small vessels pierce the tissue, perfusing it with blood. Because we have a large number of vessels of small caliber, it is impractical to model each one of them separately via Stokes or Navier–Stokes equations, as it is done in the case of large arteries. Instead, we use a PDE system that describes fluid flow in deformable, porous media (poroelastic systems), where blood vessels are modeled as pores within a deformable solid representing the tissue. Due to the fact that the local behavior is related to the systemic features of surrounding blood circulation, we couple the PDE system with a 0D simplified model, obtained by the analogy between fluid flows in hydraulic networks and currents flowing in electrical circuits. These reduced models correctly predict the systemic phenomena (like flow rate distribution and pressure pulse propagation). Our group has been particularly interested in ocular physiology, for which this multiscale interface coupling has been successfully validated against experimental measurements, including data obtained via color Doppler imaging and optical coherence tomography [17].

The PDE system of poroelasticity and the 0D lumped circuit are coupled through interface conditions that involve mean pressure values and flow rates in the adjacent neighborhood of the 3D model. The two models require very different level of details, and this is highlighted and very relevant at the interface. While the ODE focuses on mean data, the PDE requires pointwise conditions. One way to enforce continuity of pressure between the two dynamics at the interface is to assume the pressure distribution on the 3D side of the multiscale interface to be constant in space. Poiseuille's law, which relates the volumetric flow rate through the connecting resistor between the two systems with the pressure difference between the poroelasticity and circuit ends of the resistor, needs to be satisfied at the interface. Accordingly, one has to adjust the interface condition on the Biot discharge velocity, which becomes a nonlocal interface condition in this case (see the details explaining Equation 13). This set of interface conditions was introduced and analyzed in [18]. In this article, we introduce a second set of interface conditions, also driven by physical considerations, which include a much weaker constraint than what is required in [18]. More specifically, the combination of normal velocity and pressure, rather than pressure alone, is enforced to be constant in space. This translates into a natural treatment of the interface conditions similar to [5, 8], where the Navier–Stokes equations were coupled with a hydraulic network. In this paper, we compare the behavior of the solutions for the two different scenarios analytically and numerically.

## 2 Mathematical Model

### 2.1 The Local Problem—Poroelasticity

Fluid flows in deformable, porous media are modeled by systems of PDEs that describe this fluid–solid mixture as a continuum body, where the complexity of the microstructure is accounted via effective parameters. For the mathematical setting, we let $\Omega \subset {\mathbb{R}}^{3}$ be the domain occupied by the fluid–solid mixture. If x is the position vector with respect to a fixed Cartesian system, then we denote by $V\left(x,t\right)$ the representative elementary volume, which is the sum of the volumes of solid and fluid components, $V_{s}\left(x,t\right)$ and $V_{f}\left(x,t\right)$, respectively. We introduce the volumetric fractions $\phi \left(x,t\right)=\frac{V_{f}\left(x,t\right)}{V\left(x,t\right)}$, which is known as porosity, and its counterpart $\phi_{s}\left(x,t\right)=\frac{V_{s}\left(x,t\right)}{V\left(x,t\right)}$. We work under the assumption of fully saturated mixture, meaning that $\phi \left(x,t\right)+\phi_{s}\left(x,t\right)=1$, and small deformations for the homogeneous porous medium. The fully dynamical system of poroelasticity is then given by two conservation laws: the momentum balance equation for the fluid–solid mixture and the mass balance equation for the fluid, written in terms of the elastic displacement u and the fluid pressure p:

(1) $$\left\{\begin{array}{ll} \rho u_{\mathrm{tt}}-\mu_{e}\Delta u-\left(\lambda_{e}+\mu_{e}\right)\nabla \left(\nabla \cdot u\right)+\alpha \nabla p=F\left(x,t\right), & \text{in}\;\Omega \times \left(0,T\right)\\ {\left(c_{0}p+\alpha \nabla \cdot u\right)}_{t}-\nabla \cdot k\nabla p=S\left(x,t\right), & \text{in}\;\Omega \times \left(0,T\right), \end{array}\right.$$

where ρ>0 is density of the porous, permeable medium, $\lambda_{e}$, $\mu_{e}$ are the Lamé parameters, α>0 is the Biot‐Willis constant, which accounts for the pressure‐deformation coupling, and $c_{0}\geq 0$ is the constrained storage coefficient which combines the porosity of the medium and the compressibility of the fluid. Moreover, k represents the permeability, $F\left(x,t\right)$ is an elastic body force, and $S\left(x,t\right)$ is a fluid source. We remark that the term that is differentiated in time in the mass balance equation $\zeta =c_{0}p+\alpha \nabla \cdot u$ is known as the fluid content.

Mathematically, the theory of poroelasticity was initiated by M. Biot in the 1940s, rendering poroelasticity its synonymous name of Biot systems [19]. These poroelastic systems have lots of applications, including settlement of soils under load and wave propagation in fluid‐saturated porous media, and were initially inspired by geophysics and petroleum engineering problems, where they were studying fluid flow through rocks and soil. However, nowadays, these poroelastic models are used in many applications coming from biology, medicine and bioengineering, as they describe fluid flow inside cartilages, bones and engineered tissue scaffolds [20, 21, 22, 23, 24], and the eye [12, 25, 26, 27]. In most biological and biomechanical applications, the inertial effects are negligible (no acceleration terms are present), simplifying the system to the case of quasi‐static deformation:

(2) $$\left\{\begin{array}{ll} -\mu_{e}\Delta u-\left(\lambda_{e}+\mu_{e}\right)\nabla \left(\nabla \cdot u\right)+\alpha \nabla p=F\left(x,t\right) & \text{in}\;\Omega \times \left(0,T\right)\\ {\left(c_{0}p+\alpha \nabla \cdot u\right)}_{t}-\nabla \cdot k\nabla p=S\left(x,t\right) & \text{in}\;\Omega \times \left(0,T\right) \end{array}\right.$$

Another important consideration is that biological tissues have a mass density close to that of water, which means that we work under the assumption of incompressible fluid and solid constituents (solid and fluid phases can't undergo volume changes at the microscale). Mathematically, this assumption gives the following parameter simplification: $c_{0}=0$ and α=1 [28]. Therefore, the quasi‐static poroelastic system becomes

(3) $$\left\{\begin{array}{ll} -\mu_{e}\Delta u-\left(\lambda_{e}+\mu_{e}\right)\nabla \left(\nabla \cdot u\right)+\nabla p=F\left(x,t\right) & \text{in}\;\Omega \times \left(0,T\right)\\ {\left(\nabla \cdot u\right)}_{t}-\nabla \cdot k\nabla p=S\left(x,t\right) & \text{in}\;\Omega \times \left(0,T\right), \end{array}\right.$$

which can be written equivalently and more condensed in terms of the total stress of the fluid–solid mixture

(4) $$T=2\mu_{e}\epsilon \left(u\right)+\lambda_{e}\left(\nabla \cdot u\right)\;I-pI=\mu_{e}\left(\nabla u+{\left(\nabla u\right)}^{T}\right)+\lambda_{e}\left(\nabla \cdot u\right)\;I-pI$$

and the discharge velocity

(5) v=−k∇p

as

(6) $$\left\{\begin{array}{ll} \nabla \cdot T\left(u,p\right)+F\left(x,t\right)=0 & \text{in}\;\Omega \times \left(0,T\right)\\ {\left(\nabla \cdot u\right)}_{t}+\nabla \cdot v=S\left(x,t\right) & \text{in}\;\Omega \times \left(0,T\right). \end{array}\right.$$

### 2.2 The Lumped Hydraulic Circuit

**FIGURE 1:**
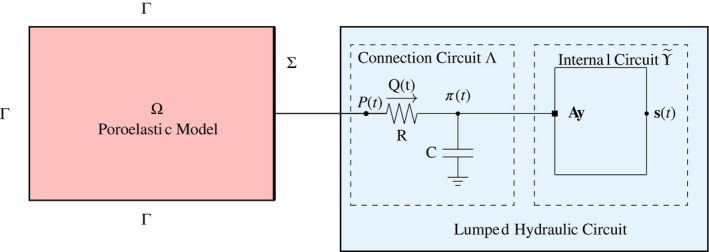
Schematics for the multiscale interface coupling of poroelasticity and lumped hydraulic circuit.

In the lumped circuit, the fluid flow through a hydraulic network is described via its analogy with a current flowing through an electrical circuit, where the active sources of voltage and current correspond to imposed pressure and flow rates, while the passive elements, like the resistances, inductances, and capacitances correspond to viscous effects, inertial effects, and vessel compliance, respectively. The circuit contains linear, time‐invariant resistors, inductors, and capacitors (RCL circuit), which correspond to assuming that the vasculature is compliant, but with small deformations, and passive. We can introduce appropriate state variables for each inductor and capacitor in the circuit [29], namely across each inductor i, we have the volumetric flow rate $Q_{i}$, and across each capacitor j, we have the pressure difference $\Pi_{j}$. The lumped hydraulic circuit ϒ (see Figure 1) is composed of

- The *internal circuit* $\tilde{ϒ}$, which contains all the lumped RCL elements that are not directly connected to the interface Σ.

We assume that $\tilde{ϒ}$ includes

- $n_{R}$ resistors with constant resistances $R_{i}>0,i=\bar{1,n_{R}}$,
- $n_{C}$ capacitors with constant capacitances $C_{i}>0,i=\bar{1,n_{C}}$,
- $n_{L}$ inductors with constant inductances $L_{i}>0,i=\bar{1,n_{L}}$.

ii The *connection* Λ, which is the bridging region between the internal circuit $\tilde{ϒ}$ and the interface Σ (Figure 1). We assume that the connection Λ consists of a resistance R and a capacitance C connected to the ground. We note that the capacitor within the connecting part Λ of the circuit is set to ground where zero pressure is assumed. Thus we write Π=π−0=π and this allows us to choose the pressure π as state variable for the capacitor C in Λ.

The vector of state variables is given by

(7) $$y\left(t\right)={\left[\pi \left(t\right),\tilde{y}\left(t\right)\right]}^{T}\in {\mathbb{R}}^{d},$$

where $\pi \left(t\right)$ is the pressure at the connecting node (see Figure 1), $\tilde{y}\left(t\right)={\left[\Pi_{1}\left(t\right),\ldots ,\Pi_{n_{C}}\left(t\right),Q_{1}\left(t\right),\ldots ,Q_{n_{L}}\left(t\right)\right]}^{T}$ is the vector of state variables for $\tilde{ϒ}$, and the dimension $d=1+n_{C}+n_{L}$.

Then the dynamics of the circuit ϒ is described by the following ODE system in $\left(0,T\right)$:

(8) $$\frac{dy\left(t\right)}{\mathrm{dt}}=A\;y\left(t\right)+s\left(t\right)+b\left(Q\left(t\right)\right),$$

where the d×d matrix A has given constant entries that depend on the circuit graph and on the values of the resistances, capacitances and inductances within the circuit. Moreover, the d×1 vector $s\left(t\right)$ represents the sources of pressure and flow rate present in $\tilde{ϒ}$. The d×1 vector $b\left(Q\left(t\right)\right)$ represents the contribution from the connection with the poroelastic Biot model and can be written as

(9) $$b\left(Q\left(t\right)\right)=\frac{Q\left(t\right)}{C}e_{1}\;\text{with}\;e_{1}={\left[1,\;0\right]}^{T}$$

where $Q=Q\left(t\right)$ is the volumetric flow rate through the connecting resistor, as shown in Figure 1, and $0\in {\mathbb{R}}^{1\times \left(d-1\right)}$.

### 2.3 Boundary, Interface, and Initial Conditions

We divide the boundary of the fluid–solid mixture domain Ω into two parts ∂Ω=Γ∪Σ, where Σ represents the portion of the boundary where the poroelastic system is coupled with the lumped circuit. For the remainder of the paper, we make the convention that boundary conditions refer to conditions imposed on Γ, while interface conditions refer to the coupling conditions imposed on the interface Σ.

#### 2.3.1 Boundary Conditions

The poroelastic system (3) described in Section 2.1 can be completed with different boundary conditions representing different physical constraints on the portion Γ of Ω. We adopt the following choices of boundary conditions, which are relevant for the applications considered. We decompose $\Gamma =\Gamma_{D,v}\cup \Gamma_{D,p}\cup \Gamma_{N}\cup \Gamma_{0}$ and assume that the following boundary conditions hold:

(10a) $$T\;n=g,\;v\cdot n=0,\;\mathrm{on}\;\Gamma_{N}\times \left(0,T\right),$$

(10b) $$\begin{array}{ccc} u=0, & p=0, & \mathrm{on}\;\Gamma_{D,p}\times \left(0,T\right), \end{array}$$

(10c) $$\begin{array}{ccc} u=0, & v\cdot n=\psi , & \mathrm{on}\;\Gamma_{D,v}\times \left(0,T\right), \end{array}$$

(10d) $$\begin{array}{ccc} \left(T\;\vec{n}\right)\cdot t_{1}=0, & \left(T\;\vec{n}\right)\cdot t_{2}=0, & \mathrm{on}\;\Gamma_{0}\times \left(0,T\right), \end{array}$$

(10e) $$\begin{array}{ccc} u\cdot n=0, & v\cdot n=0, & \mathrm{on}\;\Gamma_{0}\times \left(0,T\right), \end{array}$$

where g and ψ are given functions of space and time, n is the outward unit normal vector to the surface Γ, and $t_{i}$, with i=1,2, are the tangential unit vectors to the surface.

We remark that conditions ((10a), (10b), (10c)) are the same as those considered in [30], whereas conditions ((10d), (10e)) are instrumental for the derivation of the associated 1D model illustrated in Section 2.2.

#### 2.3.2 Interface Conditions

At the interface Σ, we consider the following conditions:

- First, we prescribe a boundary condition on Σ for the elastic part of the poroelastic medium. Here we assume that

(11) $$u\left(x,t\right)=0$$

We note that this interface condition completes the elastic problem and may be substituted with other options depending on the physical constraint experienced by the solid phase at the 3D/0D interface.

- Poiseuille's Law (the hydraulic analogue of Ohm's Law) holds for the resistor connecting the circuit to the poroelastic medium:

(12) $$Q\left(t\right)=\frac{P\left(t\right)-\pi \left(t\right)}{R},$$

linking the volumetric flow rate through the connecting resistor Q with $P\left(t\right)$ and $\pi \left(t\right)$, which are the pressures at the poroelasticity and circuit ends of the resistor, as shown in Figure 1.

- The pressure $P\left(t\right)$ at the poroelastic end of the resistor in the connection Λ has to be related to the poroelastic pressure $p\left(x,t\right)$ in order to ensure the continuity of pressure. Accordingly, we need to adjust the interface condition on the Biot discharge velocity, in order to ensure that Ohm's law holds. Therefore, we consider the following two distinct scenarios.

Case 1 **[Pointwise pressure condition (PPC)]**. We enforce the continuity of pressure in a pointwise manner, by setting

(13) $$P\left(t\right)=p\left(x,t\right),\;\text{and then we assume that}\;Q\left(t\right)=\int_{\Sigma }v\left(x,t\right)\cdot n\left(x\right)\;d\Sigma$$

Case 2 **[Integral pressure condition (IPC)]**. We enforce the continuity of pressure in an integral manner, as follows

(14) $$\begin{array}{cc} P\left(t\right)=\; & \frac{1}{m\left(\Sigma \right)}\int_{\Sigma }p\left(x,t\right)\;d\Sigma \;\text{and then we assume that}\;\\ & Kv\left(x,t\right)\cdot n=p\left(x,t\right)-\pi \left(t\right), \end{array}$$

with R>0 being the connecting resistance, and $K=R\cdot m\left(\Sigma \right)$, where $m\left(\Sigma \right)$ stands for the measure of Σ.

Remark 1 In the PPC case, the pressure distribution on the 3D side of the multiscale interface is enforced to be constant in space. This is a much stronger constraint than what is required in the IPC case, where the combination of normal velocity and pressure, rather than pressure alone, is enforced to be constant in space.

Remark 2 Since we match a 3D Biot model with a 0D lumped model, representing a much bigger region, we acknowledge that there is a big difference in the level of detail provided by these two models, which is highlighted and very relevant at the level of the interface. On one hand, the ODE requires mean data, like the pressure $\pi \left(t\right)$ and $Q\left(t\right)$, which is the volumetric flow rate through the connecting resistor. On the other hand, the Biot model requires pointwise conditions. In Case 2 we address this mismatch by introducing a natural treatment of the boundary conditions following [5, 8], where a similar approach was taken in the case of Navier–Stokes equations coupled with a hydraulic network.

#### 2.3.3 Initial Conditions

The problem is completed by the following initial conditions:

(15) $$u\left(x,0\right)=u_{0}\left(x\right)\;\text{for}\;x\in \Omega ,\;y\left(0\right)=y_{0}$$

with $u_{0}\left(x\right)$ and $y_{0}$ given.

## 3 Physical Energy

The elastic energy associated with the Biot system is given by

(16) $${ℰ}_{\Omega }\left(u\right)=\frac{\lambda }{2}∥\nabla \cdot u{∥}_{L^{2}\left(\Omega \right)}^{2}+\mu ∥E\left(u\right){∥}_{L^{2}\left(\Omega \right)}^{2},$$

where the tensor $E\left(u\right)$ is the symmetric part of the gradient of the vector field u, defined as $E\left(u\right)=1/2\left(\nabla u+{\left(\nabla u\right)}^{T}\right).$

The energy stored in the capacitors and inductors within the circuit ϒ has the following formula:

(17) $${ℰ}_{ϒ}\left(y\right)=\frac{C\pi^{2}}{2}+\sum_{i=1}^{n_{C}}\frac{C_{i}\Pi_{i}^{2}}{2}+\sum_{i=1}^{n_{L}}\frac{L_{i}Q_{i}^{2}}{2}.$$

Alongside the energies, we introduce the notation for the Biot viscous dissipation $D_{\Omega }\left(p\right)$ and the circuit dissipation $D_{ϒ}\left(y\right)$:

(18) $$D_{\Omega }\left(p\right)=∥k^{1/2}\nabla p{∥}_{L^{2}\left(\Omega \right)}^{2}$$

(19) $$D_{ϒ}\left(y\right)=\left(\mathrm{By}\right)\cdot y,$$

where B is defined below in (31).

Case 1 [PPC]. We remark that the mathematical problem ((3), (4), (5), (6), (7), (8)) is characterized by an energy identity that captures the physical phenomena governing the dynamics of the coupled system:
(20)

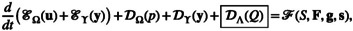

where ${ℰ}_{\Omega }\left(u\right)$, ${ℰ}_{ϒ}\left(y\right)$, $D_{\Omega }\left(p\right)$ and $D_{ϒ}\left(y\right)$ represent the energies and dissipation functionals in Ω and ϒ, which were defined above. Notably, $D_{\Lambda }\left(Q\right)$ is the dissipation in the connection Λ, and is given by

$$D_{\Lambda }\left(Q\right)={\mathrm{RQ}}^{2}\left(t\right).$$

The details for obtaining (20) are similar to the ones described below for Case 2 [IPC] and can also be found in [18].

Case 2 [IPC]. The multiscale system ((3), (4), (5), (6), (7), (8)) is characterized by an energy balance of the form:
(21)

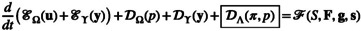

where ${ℰ}_{\Omega }\left(u\right)$, ${ℰ}_{ϒ}\left(y\right)$, $D_{\Omega }\left(p\right)$ and $D_{ϒ}\left(y\right)$ represent the energies and dissipation functionals in Ω and ϒ, and $D_{\Lambda }\left(\pi ,p\right)$ is the dissipation in the connection Λ. The formulas for the two dissipation terms are provided in (18) and (19). The source ℱ depends on the volumetric and boundary forcing terms and it is given by

$$ℱ\left(S,F,g,s\right)={ℱ}_{\Omega }\left(F,S;u,p\right)+{ℱ}_{\partial \Omega }\left(g,\psi ;u,p\right)+{ℱ}_{ϒ}\left(s;y\right),$$

with ${ℱ}_{\Omega }\left(F,S;u,p\right)$, ${ℱ}_{\partial \Omega }\left(g,\psi ;u,p\right)$, and ${ℱ}_{ϒ}\left(s;y\right)$ given below in (26), (27), and (30). Crucially, we will show that $D_{\Lambda }\left(\pi ,p\right)\geq 0$. In order to obtain the energy identity (21), we begin by focusing on the Biot problem. Using multipliers $u_{t}$ and p for the linear momentum balance equation and the mass balance equation, respectively, and integrating by parts in space, we obtain

(22) $$\begin{array}{c} \int_{\Omega }\left(\lambda \nabla \cdot uI..\nabla u_{t}+2\mu E\left(u\right)..\nabla u_{t}\right)\;d\Omega -\int_{\partial \Omega }\left(\mathrm{Tn}\right)\cdot u_{t}\;\mathrm{dS}\\ -\int_{\Omega }\nabla \cdot \left(k\nabla p\right)p\;d\Omega =\int_{\Omega }F\left(x,t\right)\cdot u_{t}\;d\Omega +\int_{\Omega }S\left(x,t\right)p\;d\Omega \end{array}$$

where in the above equation we used the notation “..” to represent the Frobenius inner product of two matrix fields.

We integrate by parts in space in the last two terms on the left hand side of (22) and obtain
(23)

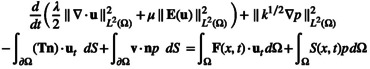




Recalling the boundary and interface conditions, (23) simplifies to
(24)

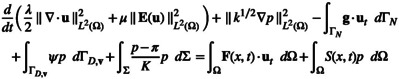




Recalling the definition of $P\left(t\right)$ and K

$$P\left(t\right)=\frac{1}{m\left(\Sigma \right)}\int_{\Sigma }p\left(x,t\right)\;d\Sigma \;\text{and}\;K=R\cdot m\left(\Sigma \right),$$

we obtain the following energy identity for the Biot system

(25) $$\begin{array}{cc} \frac{d}{\mathrm{dt}}{ℰ}_{\Omega }\left(u\right)+D_{\Omega }\left(p\right)=\; & {ℱ}_{\Omega }\left(F,S;u,p\right)+{ℱ}_{\partial \Omega }\left(g,\psi ;u,p\right)\\ & +\frac{1}{R}P\left(t\right)\pi \left(t\right)-\frac{1}{K}∥p{∥}_{L^{2}\left(\Sigma \right)}^{2} \end{array}$$

where the elastic energy ${ℰ}_{\Omega }\left(u\right)$ and the viscous dissipation $D_{\Omega }\left(p\right)$ are defined in (16) and (18), and the forcing terms ${ℱ}_{\Omega }\left(F,S;u,p\right)$ and ${ℱ}_{\partial \Omega }\left(g,\psi ;u,p\right)$ are given by:

(26) $${ℱ}_{\Omega }\left(F,S;u,p\right)=\int_{\Omega }F\cdot u_{t}\;d\Omega +\int_{\Omega }S\;p\;d\Omega$$

(27) $${ℱ}_{\partial \Omega }\left(g,\psi ;u,p\right)=\int_{\Gamma_{N}}g\cdot u_{t}\;d\Gamma_{N}-\int_{\Gamma_{D,v}}\mathrm{\psi p}\;d\Gamma_{D,v}.$$

Then we consider the ODE system describing the dynamics of the circuit. We define the following diagonal matrix whose positive entries are given by the capacitances and inductances present in the lumped circuit

(28) $$U≔\mathrm{diag}\left(\left[C,C_{1},\ldots ,C_{n_{C}},L_{1},\ldots ,L_{n_{L}}\right]\right).$$

Using multiplier Uy in system (8), we obtain the following identity

(29) $$\frac{d{ℰ}_{ϒ}\left(y\right)}{\mathrm{dt}}+D_{ϒ}\left(y\right)={ℱ}_{ϒ}\left(s;y\right)+\left(\mathrm{Uy}\right)\cdot b\left(Q\left(t\right)\right),$$

where the circuit energy ${ℰ}_{ϒ}\left(y\right)$ and dissipation $D_{ϒ}\left(y\right)$ are defined in (17) and (19), respectively, and the forcing term within the circuit (due to sources of flow rate and pressure, analogous to current and voltage sources) is given by

(30) $${ℱ}_{ϒ}\left(s;y\right)=\left(\mathrm{Uy}\right)\cdot s$$

${ℰ}_{ϒ}\left(y\right)$ The circuit dissipation $D_{ϒ}\left(y\right)$ depends on the matrix B, which is given by

(31) B=−UA.

Since we are considering linear time‐invariant elements, the circuit is passive and, consequently, the matrix B is positive definite [29], implying that the functional $D_{ϒ}\left(y\right)$ represents a dissipation of energy within the circuit elements.

Now, using the definition (28) of the matrix U and the definition (9) of the vector b, we obtain that

$$\left(\mathrm{Uy}\right)\cdot b\left(Q\left(t\right)\right)=\pi \left(t\right)Q\left(t\right)$$

and therefore (29) becomes

(32) $$\frac{d{ℰ}_{ϒ}\left(y\right)}{\mathrm{dt}}+D_{ϒ}\left(y\right)={ℱ}_{ϒ}\left(s;y\right)+\pi \left(t\right)Q\left(t\right).$$

Combining (25) and (32) we obtain

(33) $$\begin{array}{c} \frac{d}{\mathrm{dt}}\left(E_{\Omega }\left(u\right)+E_{ϒ}\left(y\right)\right)+D_{\Omega }\left(p\right)+D_{ϒ}\left(y\right)=\\ \;F_{\Omega }\left(F,S;u,p\right)+F_{\partial \Omega }\left(g,\psi ;u,p\right)+F_{ϒ}\left(s;y\right)+Q\left(t\right)\pi \left(t\right)+\frac{1}{R}P\left(t\right)\pi \left(t\right)-\frac{1}{K}∥p{∥}_{L^{2}\left(\Sigma \right)}^{2} \end{array}$$

Finally, using (12) in (33), we obtain the following energy identity for the fully coupled system

(34) $$\begin{array}{c} \frac{d}{\mathrm{dt}}\left(E_{\Omega }\left(u\right)+E_{ϒ}\left(y\right)\right)+D_{\Omega }\left(p\right)+D_{ϒ}\left(y\right)+D_{\Lambda }\left(\pi ,p\right)\\ =F_{\Omega }\left(F,S;u,p\right)+F_{\partial \Omega }\left(g,\psi ;u,p\right)+F_{ϒ}\left(s;y\right) \end{array}$$

where

(35) $$D_{\Lambda }\left(\pi ,p\right)=\frac{1}{R}\pi^{2}\left(t\right)+\frac{1}{K}∥p{∥}_{L^{2}\left(\Sigma \right)}^{2}-\frac{2P\left(t\right)\pi \left(t\right)}{R}=\frac{1}{K}\int_{\Sigma }{\left(p\left(x,t\right)-\pi \left(t\right)\right)}^{2}\;d\Sigma$$

is the dissipation due to the resistive connection between the Biot region and the internal circuit.

Remark 3 Note that both interface conditions [PPC] and [IPC] render an energy identity where the connection between the circuit and the poroelasticity introduces a damping mechanism.

## 4 Prior Results

While analytical and numerical studies for fluid flows through deformable porous media in biological models are available in the literature, including [11, 18, 30, 31, 32, 33, 34, 35, 36, 37, 38, 39, 40, 41, 42, 43], there are only a few recent papers that investigate poroelastic systems coupled with 0D lumped models accounting for systemic features. The multiscale interface 3D‐OD coupling brings new challenges and implicitly calls for novel theoretical and computational approaches.

Local well‐posedness analysis for the multiscale interface coupling ((3), (4), (5), (6), (7), (8)) along with this set of interface conditions ((11), (12)) was provided recently in [44]. The multiscale interface coupling ((3), (4), (5), (6), (7), (8)) along with this set of interface conditions ((11), (12)) was investigated numerically in authors' recent paper [18]. We remark that the interface conditions presented in Section 2.3 – Case 1 (PPC) are nonlocal for the Biot discharge velocity v. Even though similar nonlocal interface conditions arise in 3D/0D models involving the Stokes/Navier–Stokes equations, the mathematical challenges that these conditions bring for deformable porous media are very different. Unlike the Stokes/Navier–Stokes case, in deformable porous media, the discharge velocity v is not solenoidal, thereby yielding less control over its normal component at the interface, whose integral appears in the interface conditions. This difference requires novel theoretical and computational strategies.

The numerical solution of problems involving 3D/0D interface coupling has drawn particular attention in the context of S/NS equations and lumped hydraulic circuits [1, 2, 3, 4, 6, 7, 8, 9, 10] in recent years. The majority of these methods rely on decoupling the 3D and 0D problems via a fixed‐point mapping, where the interface conditions are transformed into prescribed boundary conditions; by doing so, a mismatch is introduced among the interface variables, which is resolved by iterating between the 3D and 0D problems till convergence is achieved. This approach has a considerable advantage in that the decoupled PDE problem presents standard boundary conditions, thus making the use of prior numerical studies on the PDE system straightforward. Inspired by the above‐mentioned numerical studies on 3D/0D coupling for S/NS equations, we conducted a similar study where a fixed‐point mapping was implemented to solve the multiscale interface coupling ((3), (4), (5), (6), (7), (8)) in [18]. For the sake of simplicity, a 1D version of the poroelastic system was considered, so that an exact solution can be computed analytically and compared with its numerical approximation. In the fixed‐point mapping, also referred to as *PQP* iterations, in any discrete time interval $\left(t^{n},t^{n+1}\right)$, with $t^{n}=n\Delta t$, the poroelastic system is solved with a given interface pressure ($P_{j}$) to then compute a new interface flow rate ($Q_{j+1}$) that can be used as an input for the lumped circuit and obtain an updated value for the interface pressure ($P_{j+1}$) and then continue till convergence.

Mathematically, the map is given by $P_{j+1}=\varphi \left(P_{j}\right)$, where $\varphi \left(s\right)=\gamma_{1}\left(\Delta t,z\right)-\gamma_{2}\left(\Delta t,z\right)s$, z being the vector of model parameters (i.e., k, R, C, and the lumped parameters in $\tilde{ϒ}$). The linearity of φ is not surprising, since the problem is linear. Unexpectedly, however, its coefficients depend nonlinearly on Δt, which is important since $|\varphi^{\prime }\left(s\right)|=|\gamma_{2}\left(\Delta t,z\right)|<1$ is a sufficient condition for the *PQP* iterations to converge. When $\gamma_{2}\left(\Delta t,z\right)$ is graphed as a function of Δt for a specific set of parameters z, one can see that sufficient condition for the iterations to converge is *not* satisfied as Δt→0 [18].

Numerical simulations performed using a Backward Euler discretization in time for both the PDE and ODE systems, in order to ensure stability of the two problems when solved separately, and a Finite Element approximation in primal variables for the space discretization of the 1D poroelastic system, confirm that the *PQP* iterations do not converge if Δt is less than $10^{-4},$ with associated blow‐up in the numerical solution. This blow‐up is clearly a numerical artifact, since it is not displayed by the exact analytical solution, and it is due to the mismatch among the interface variables at each iteration. Specifically, the discrete counterpart of the dissipation in the physical energy of the system does not have a definite sign [18], and therefore can give rise to spurious, destabilizing interface forces unless restrictive conditions are imposed on the model parameters z to guarantee convergence. Such conditions, however, may unacceptably limit the usefulness and impact of our work in the study of tissue perfusion. The numerical experiments in [18] show a loss of convergence if the resistance R in the 1D/0D connection becomes too large and/or the permeability k of the Biot model becomes too small and/or the elastic parameters $\mu_{e}$ and $\lambda_{e}$ in the Biot model become too large. In application (like optic nerve head perfusion), large values of R may occur due to vessel narrowing or partial vessel collapse [45], decreased k may occur due to ischemic damage [46], and increased $\mu_{e}$ and $\lambda_{e}$ may occur due to tissue stiffening and remodeling [47]. If one aims to provide a rational approach to adjust modifiable variables in the system to maintain appropriate levels of tissue perfusion and prevent the aforementioned pathological conditions from happening, one can't a priori limit themselves to a numerical approach that only allows to consider healthy scenarios. Accordingly, in [18] the multiscale interface coupling ((3), (4), (5), (6), (7), (8)) was solved using an operator splitting algorithm, where the 3D and 0D communicate through initial conditions. Unconditional stability for the energy‐based operator splitting method with respect to the size of the time discretization step was proved, which was a consequence of the fact that the splitting step did not disrupt the energy balance holding at the continuous level, ensuring in particular that the dissipation mechanism has a nonnegative sign. Based on this consideration, we also investigated [18] how dissipation was handled by the method based on functional iterations and its connection with failure of convergence. In addition to the solution of 1D–0D coupled problems, simulations were also presented for the full 3D–0D case. The numerical results supported the theoretical findings and, interestingly, confirmed that decreasing the time step size did not always guarantee convergence for the functional iterations.

## 5 Operator Splitting Algorithm

Inspired by the results from [18] presented in the previous section, we use an operator splitting approach to numerically approximate the solutions for the multiscale interface coupling with both sets of interface conditions.

The operator splitting method consists in splitting the coupled system into separate parts and defining a communication among the parts through the initial conditions [48]. If one designs the operator splitting algorithm in such a way as to maintain at the discrete level the energy properties of the coupled system, unconditional stability with respect to the choice of the time step may be achieved without the need of subiterations among the substeps [1, 49, 50, 51, 52, 53]. Inspired by the energy identities obtained in Section 3, we split the multiscale interface coupling by treating the interface conditions together with the poroelastic model in a first substep, thus implicitly ensuring mass conservation and pressure continuity at the interface, and solving the internal part of the circuit ϒ in the second substep.

### 5.1 IPC

For any n≥0, the proposed operator splitting method consists of solving sequentially the following two steps:

Step 1 Given $u^{n}$ and $y^{n}=\left[\pi^{n},{\tilde{y}}^{n}\right]$, solve:

(36) $$\begin{array}{ll} \;\nabla \cdot T\left(u,p\right)+F\left(x,t\right)=0\; & \text{in}\;\Omega \times \left(t^{n},t^{n+1}\right)\\ {\left(\nabla \cdot u\right)}_{t}-\nabla \cdot k\nabla p=S\left(x,t\right)\; & \text{in}\;\Omega \times \left(t^{n},t^{n+1}\right)\\ \;\frac{\mathrm{d\pi}}{\mathrm{dt}}=\frac{Q\left(t\right)}{C}\; & \text{in}\;\left(t^{n},t^{n+1}\right) \end{array}$$

with the boundary conditions:

(37a) $$\begin{array}{ccc} T\;n=g, & v\cdot n=0, & \mathrm{on}\;\Gamma_{N}\times \left(t^{n},t^{n+1}\right), \end{array}$$

(37b) $$\begin{array}{ccc} u=0, & p=0, & \mathrm{on}\;\Gamma_{D,p}\times \left(t^{n},t^{n+1}\right), \end{array}$$

(37c) $$\begin{array}{ccc} u=0, & v\cdot n=\psi , & \mathrm{on}\;\Gamma_{D,v}\times \left(t^{n},t^{n+1}\right), \end{array}$$

(37d)

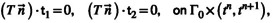

(37e) $$\begin{array}{ccc} u\cdot n=0, & v\cdot n=0, & \mathrm{on}\;\Gamma_{0}\times \left(t^{n},t^{n+1}\right), \end{array}$$

and the interface conditions:

(38) $$u=0\;\mathrm{on}\;\Sigma \times \left(t^{n},t^{n+1}\right)$$

(39) $$Kv\left(x,t\right)\cdot n=p\left(x,t\right)-\pi \left(t\right)\;\mathrm{on}\;\Sigma \times \left(t^{n},t^{n+1}\right)$$

(40) $$\frac{1}{m\left(\Sigma \right)}\int_{\Sigma }p\left(x,t\right)dx=P\left(t\right)\;\mathrm{on}\;\left(t^{n},t^{n+1}\right)$$

(41) $$Q\left(t\right)=\frac{P\left(t\right)-\pi \left(t\right)}{R}\;\mathrm{on}\;\left(t^{n},t^{n+1}\right)$$

and initial conditions

(42) $$u\left(t^{n}\right)=u^{n}\;\text{in}\;\Omega \;\text{and}\;\pi \left(t^{n}\right)=\pi^{n}.$$

Then we set $u^{n+1/2}=u\left(t^{n+1}\right)$, $p^{n+1/2}=p\left(t^{n+1}\right)$, and $y^{n+1/2}=\left[\pi \left(t^{n+1}\right),{\tilde{y}}^{n}\right]$.

Step 2 Given $y^{n+1/2}$, solve

(43) $$\frac{dy}{\mathrm{dt}}=\mathrm{Ay}+s\left(t\right)\;\text{in}\;\left(t^{n},t^{n+1}\right)\;\text{with initial condition}\;y\left(t^{n}\right)=y^{n+1/2}.$$

Then set $y^{n+1}=y\left(t^{n+1}\right)$. Since we do not update u and p in this step, we also have $u^{n+1}=u^{n+1/2}$ and $p^{n+1}=p^{n+1/2}$.

Remark 4 Note that the order of the two steps could be reversed. One could solve the nonlinear ODE and use this solution as initial data for the system in Step 1.

We have the following theorem regarding the stability of the operator splitting algorithm:

Theorem 1 (Stability of operator splitting method). *The operator splitting method illustrated above for the solution of the multiscale interface coupled problem* ((3), (4), (5), (6), (7), (8)) *with interface conditions* ((11), (12)) *and without external forcing is unconditionally stable with respect to the choice of the time discretization step* Δt.

First, we write the energy estimates associated with the problems introduced in the two steps separately and show that the operator splitting method does not disrupt the energy balance (34) that holds at the continuous level. This provides an a priori bound on the solution that ensures the desired unconditional stability. To clarify the notation, we will use superscripts $\cdot^{I}$ and $\cdot^{\mathrm{II}}$ to distinguish between the unknown variables involved in the two substeps of the operator splitting method.

We begin by considering the system introduced in Step 1 of the operator splitting method. Similarly to Section 3, we obtain the following energy identity

(44) $$\frac{d}{\mathrm{dt}}{ℰ}_{\Omega }\left(u^{I}\right)+D_{\Omega }\left(p^{I}\right)={ℱ}_{\Omega }\left(g,\psi ;u^{I},p^{I}\right)-P^{I}\left(t\right)Q^{I}\left(t\right)$$

where the elastic energy, viscous dissipation, and forcing term are given by

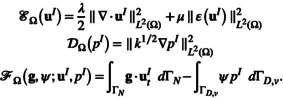




Step 1 also includes the following ODE

$$\frac{dy^{I}}{\mathrm{dt}}=b\left(Q^{I}\left(t\right)\right)={\left[\frac{Q^{I}\left(t\right)}{C},0\right]}^{T},$$

which is equivalent to the following scalar ODE for $\pi^{I}$:

$$\frac{d\pi^{I}}{\mathrm{dt}}=\frac{Q^{I}\left(t\right)}{C}.$$

The associated energy estimate is given by

(45) $$\frac{C}{2}\frac{d}{\mathrm{dt}}{\left(\pi^{I}\right)}^{2}=\pi^{I}Q^{I}.$$

Combining (44) with (45), we obtain the energy identity for the coupled system present in Step 1:

(46) $$\frac{d}{\mathrm{dt}}\left[{ℰ}_{\Omega }\left(u^{I}\right)+\frac{C}{2}{\left(\pi^{I}\right)}^{2}\right]+D_{\Omega }\left(p^{I}\right)+D_{\Lambda }\left(\pi^{I},p^{l}\right)={ℱ}_{\Omega }\left(g,\psi ;u^{I},p^{I}\right)$$

with

(47) $$D_{\Lambda }\left(\pi^{I},p^{I}\right)=\frac{1}{K}\int_{\Sigma }{\left(p^{I}\left(x,t\right)-\pi^{I}\left(t\right)\right)}^{2}\;d\Sigma$$

We emphasize here that the choice to include the connecting part Λ of the lumped circuit within Step 1 along with the poroelastic problem ensures that the numerical counterpart of $D_{\Lambda }$ is nonnegative, therefore preserving the dissipative nature that this term has at the continuous level. Furthermore, in the absence of external forcing terms, that is, ${ℱ}_{\Omega }\left(g,\psi ;u^{I},p^{I}\right)=0$, it follows that for n≥0,

(48) $$\left[{ℰ}_{\Omega }\left(u^{I}\left(t^{n+1}\right)\right)+\frac{C}{2}{\left(\pi^{I}\left(t^{n+1}\right)\right)}^{2}\right]\leq \left[{ℰ}_{\Omega }\left(u^{I}\left(t^{n}\right)\right)+\frac{C}{2}{\left(\pi^{I}\left(t^{n}\right)\right)}^{2}\right].$$

Additionally, since we are not updating the variables $\Pi_{i}$ and $Q_{i}$ in this step, we have $\Pi_{i}^{I}\left(t^{n+1}\right)=\Pi_{i}^{I}\left(t^{n}\right)$ and $Q_{i}^{I}\left(t^{n+1}\right)=Q_{i}^{I}\left(t^{n}\right)$ or, equivalently, ${\tilde{y}}^{I}\left(t^{n+1}\right)={\tilde{y}}^{I}\left(t^{n}\right)$. This implies that for n≥0, we have the following inequality

(49) $$\left[{ℰ}_{\Omega }\left(u^{I}\left(t^{n+1}\right)\right)+{ℰ}_{ϒ}\left(y^{I}\left(t^{n+1}\right)\right)\right]\leq \left[{ℰ}_{\Omega }\left(u^{I}\left(t^{n}\right)\right)+{ℰ}_{ϒ}\left(y^{I}\left(t^{n}\right)\right)\right].$$

Now we consider Step 2 of the operator splitting method, where we solve the following ODE system:

(50) $$\frac{dy^{\mathrm{II}}}{\mathrm{dt}}={\mathrm{Ay}}^{\mathrm{II}}+s\left(t\right).$$

Using multiplier ${\mathrm{Uy}}^{\mathrm{II}}$ in the circuit ODE (50) we obtain the following identity

(51) $$\frac{d{ℰ}_{ϒ}\left(y^{\mathrm{II}}\right)}{\mathrm{dt}}+D_{ϒ}\left(y^{\mathrm{II}}\right)={ℱ}^{\mathrm{II}}\left(s;y^{\mathrm{II}}\right),$$

where

(52) $${ℰ}_{ϒ}\left(y^{\mathrm{II}}\right)=\frac{C{\left(\pi^{\mathrm{II}}\right)}^{2}}{2}+\sum_{i=1}^{n_{C}}\frac{C_{i}{\left(\Pi_{i}^{\mathrm{II}}\right)}^{2}}{2}+\sum_{i=1}^{n_{L}}\frac{L_{i}{\left(Q_{i}^{\mathrm{II}}\right)}^{2}}{2}$$

is the energy stored in the capacitors and inductors within the circuit ϒ. The dissipation in the circuit is given by

$$D_{ϒ}\left(y^{\mathrm{II}}\right)=\left({\mathrm{By}}^{\mathrm{II}}\right)\cdot y^{\mathrm{II}},$$

where the matrix B is defined as in (31) and the forcing term takes the following form:

$${ℱ}^{\mathrm{II}}\left(s;y^{\mathrm{II}}\right)=\left({\mathrm{Uy}}^{\mathrm{II}}\right)\cdot s\left(t\right).$$

Hence, in the absence of external forcing terms, that is, ${ℱ}^{\mathrm{II}}\left(s;y^{\mathrm{II}}\right)=0$, and since the matrix B is positive definite (see Section 3), it follows that

(53) $${ℰ}_{ϒ}\left(y^{\mathrm{II}}\left(t^{n+1}\right)\right)\leq {ℰ}_{ϒ}\left(y^{\mathrm{II}}\left(t^{n}\right)\right).$$

Again, since we are not updating the variables u and p in this step, we have $u^{\mathrm{II}}\left(t^{n+1}\right)=u^{\mathrm{II}}\left(t^{n}\right)$ and $p^{\mathrm{II}}\left(t^{n+1}\right)=p^{\mathrm{II}}\left(t^{n}\right)$. In particular, this means that

(54) $${ℰ}_{\Omega }\left(u^{\mathrm{II}}\left(t^{n+1}\right)\right)={ℰ}_{\Omega }\left(u^{\mathrm{II}}\left(t^{n}\right)\right),$$

and thus we have that for n≥0, the following inequality is true:

(55) $${ℰ}_{\Omega }\left(u^{\mathrm{II}}\left(t^{n+1}\right)\right)+{ℰ}_{ϒ}\left(y^{\mathrm{II}}\left(t^{n+1}\right)\right)\leq {ℰ}_{\Omega }\left(u^{\mathrm{II}}\left(t^{n}\right)\right)+{ℰ}_{ϒ}\left(y^{\mathrm{II}}\left(t^{n}\right)\right).$$

Now we recall that the initial conditions for Step 2 are the solutions of Step 1, meaning that

$$\begin{array}{c} y^{\mathrm{II}}\left(t^{n}\right)=y^{n+1/2}=y^{I}\left(t^{n+1}\right),\\ u^{\mathrm{II}}\left(t^{n}\right)=u^{n+1/2}=u^{I}\left(t^{n+1}\right),\;p^{\mathrm{II}}\left(t^{n}\right)=p^{n+1/2}=p^{I}\left(t^{n+1}\right). \end{array}$$

Thus combining (55) with (49) we obtain that for n≥0, we have

(56a) $${ℰ}_{\Omega }\left(u^{\mathrm{II}}\left(t^{n+1}\right)\right)+{ℰ}_{ϒ}\left(y^{\mathrm{II}}\left(t^{n+1}\right)\right)\leq {ℰ}_{\Omega }\left(u^{\mathrm{II}}\left(t^{n}\right)\right)+{ℰ}_{ϒ}\left(y^{\mathrm{II}}\left(t^{n}\right)\right)$$

(56a) $$={ℰ}_{\Omega }\left(u^{I}\left(t^{n+1}\right)\right)+{ℰ}_{ϒ}\left(y^{I}\left(t^{n+1}\right)\right)\leq {ℰ}_{\Omega }\left(u^{I}\left(t^{n}\right)\right)+{ℰ}_{ϒ}\left(y^{I}\left(t^{n}\right)\right).$$

The chain of inequalities given in (56a) shows that the physical energies ${ℰ}_{\Omega }$ and ${ℰ}_{ϒ}$ provide norms for the solution that, in the absence of external forcing, are bounded a priori by the initial conditions regardless of the choice of the time discretization step Δt, therefore concluding the proof of the theorem.

### 5.2 PPC

The operator splitting algorithm in this case is very similar to the one presented above. The only difference is represented by the interface conditions in Step 1. Instead of (38), we consider the following interface conditions:

(57) $$\begin{array}{ll} \;u=0\; & \mathrm{on}\;\Sigma \times \left(t^{n},t^{n+1}\right)\\ \int_{\Sigma }v\left(x,t\right)\cdot n\left(x\right)dx=Q\left(t\right)\; & \mathrm{on}\;\left(t^{n},t^{n+1}\right)\\ \;p\left(x,t\right)=P\left(t\right)\; & \mathrm{on}\;\Sigma \times \left(t^{n},t^{n+1}\right)\\ \;Q\left(t\right)=\frac{P\left(t\right)-\pi \left(t\right)}{R}\; & \mathrm{on}\;\left(t^{n},t^{n+1}\right). \end{array}$$

Following the ideas introduced in the previous subsection, we obtain again that.

Theorem 2 (Stability of operator splitting method). *The operator splitting method illustrated above for the solution of the multiscale interface coupled problem* ((3), (4), (5), (6), (7), (8)) *with interface conditions* ((11), (12)) *and without external forcing is unconditionally stable with respect to the choice of the time discretization step* Δt.

The detailed proof of this theorem can be found in [18].

## 6 Formulation of the 1D–0D BIOT Coupled Problem

**FIGURE 2:**
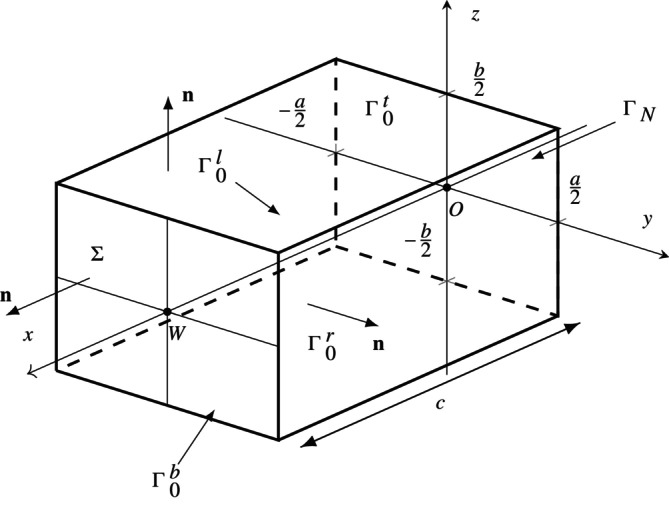
The three‐dimensional domain Ω the geometrical notation. The domain boundary is $\partial \Omega =\Gamma_{N}\cup \Gamma_{0}\cup \Sigma$, with $\Gamma_{0}=\Gamma_{0}^{t}\cup \Gamma_{0}^{b}\cup \Gamma_{0}^{l}\cup \Gamma_{0}^{r}$ representing the lateral surface. The symbol n is the outward unit normal vector on ∂Ω. The dimensions along the axes x, y and z are c, a and b, respectively, so that the area of the surface is equal to ab. W is the point at which the lumped hydraulic circuit ϒ is connected to the domain Ω.

Now we introduce a 3D setting for poroelasticity that supports an 1D motion in the body Ω. Let $\Omega =\left(0,c\right)\times \left(-a/2,a/2\right)\times \left(-b/2,b/2\right)$. Recall that the boundary ∂Ω is split into three components, $\Gamma_{N},\Gamma_{0}$ and Σ. $\Gamma_{N}$ is the plane at x=0, Σ is the plane at x=c and $\Gamma_{0}=\Gamma_{0}^{t}\cup \Gamma_{0}^{b}\cup \Gamma_{0}^{l}\cup \Gamma_{0}^{r}$ is the union of the lateral sides of Ω. The hydraulic circuit is connected to Σ at the point W, of coordinates $\left(0,0,c\right)$. We will focus on the [IPC] interface conditions, while noting that in the simplified 1D–0D case both interface conditions give rise to the same model, as it can be seen below (Figure 2).

We assume that u is a vector consisting of only the x‐component and that both u and p are functions of only x and t. Hence

$$\begin{array}{c} v=-k\nabla p={\left[-k\frac{\partial p\left(x,t\right)}{\partial x},0,0\right]}^{T}\\ T\left(u,p\right)=\left[\begin{array}{ccc} \left(\lambda +2\mu \right)\frac{\partial u\left(x,t\right)}{\partial x}-p\left(x,t\right) & 0 & 0\\ 0 & \lambda \frac{\partial u\left(x,t\right)}{\partial x}-p\left(x,t\right) & 0\\ 0 & 0 & \lambda \frac{\partial u\left(x,t\right)}{\partial x}-p\left(x,t\right) \end{array}\right] \end{array}$$

On $\Gamma_{N}$ and Σ we enforce the following boundary conditions

$$\begin{array}{c} \mathrm{Tn}=g\;\mathrm{on}\;\Gamma_{N}\times \left(0,T\right)\\ Kv\cdot n=pn-\pi \;\mathrm{on}\;\Sigma \times \left(0,T\right), \end{array}$$

where $K=\mathrm{Rm}\left(\Sigma \right)$. It is clear from the definitions of T and v that the homogeneous boundary conditions on $\Gamma_{0}$, (10d) and (10e), are satisfied.

We can now reformulate the 1D Biot model in our 3D domain. The following equations hold for all $\left(x,t\right)\in \left(0,c\right)\times \left(0,T\right)$:

(58) $$\begin{array}{r} \frac{\partial }{\partial t}\left(\frac{\partial u\left(x,t\right)}{\partial x}\right)-\frac{\partial v\left(x,t\right)}{\partial x}=S\\ -\frac{\partial T\left(u,p\right)}{\partial x}=f\\ v\left(x,t\right)=-k\frac{\partial p\left(x,t\right)}{\partial x}\\ T\left(u,p\right)=\left(\lambda +2\mu \right)\frac{\partial u\left(x,t\right)}{\partial x}-p\left(x,t\right) \end{array}$$

Alongside these equations we have the following boundary conditions at x=0 for $t\in \left(0,T\right)$:

(59) $$\begin{array}{r} \left(\lambda +2\mu \right)\frac{\partial u\left(0,t\right)}{\partial x}-p\left(0,t\right)=g\left(t\right)\\ -k\frac{\partial p\left(x,0\right)}{\partial x}=0 \end{array}$$

and interface conditions at x=c for $t\in \left(0,T\right)$:

(60) $$\begin{array}{r} P\left(t\right)=\frac{1}{\mathrm{ab}}\int_{-a/2}^{a/2}\int_{-b/2}^{b/2}p\left(c,t\right)\text{dydz}\\ u\left(c,t\right)=0\\ \left(\mathrm{Rab}\right)v\left(c,t\right)=p\left(c,t\right)-\pi \left(t\right). \end{array}$$

We complete the system with the following initial condition $\partial u\left(x,0\right)=\chi \left(x\right)$, where χ is a given function.

**FIGURE 3:**
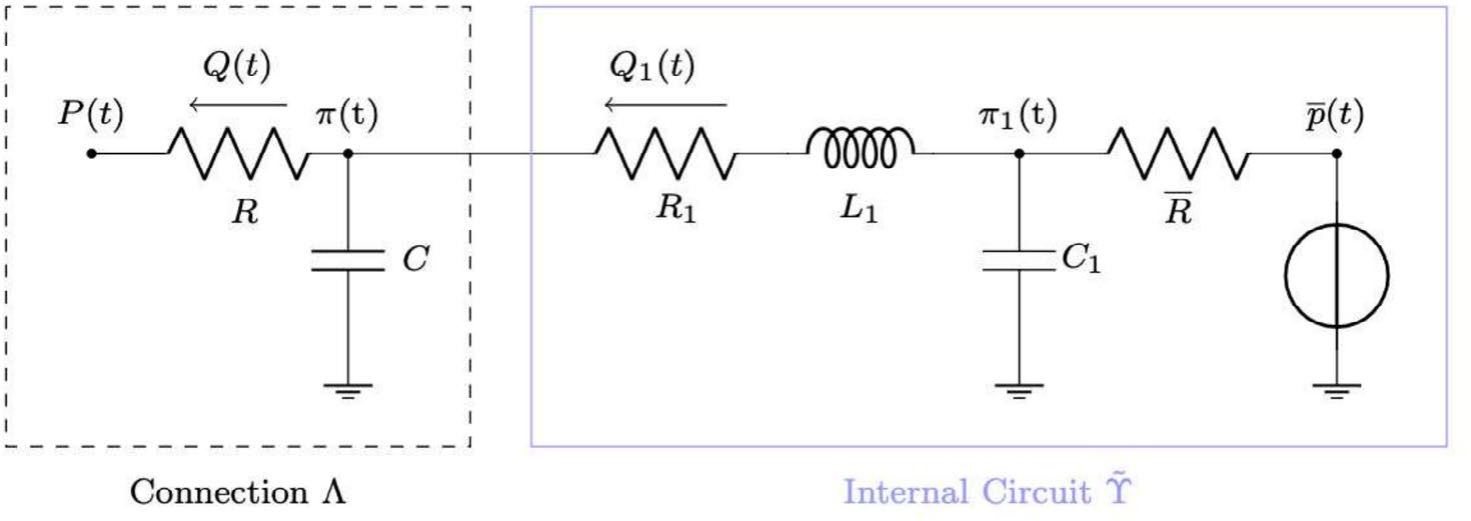
Schematic for the simplified circuit considered.

Next, we introduce the lumped hydraulic circuit that is coupled with the 1D Biot model, illustrated in Figure 3. The solution state $y\left(t\right)$ is defined as

(61) $$y\left(t\right)=\left[\pi \left(t\right),\pi_{1}\left(t\right),Q_{1}\left(t\right)\right],$$

where $\pi \left(t\right)$ is the first component of the state $y\left(t\right)$ that connects to the Biot system and $\pi_{1}\left(t\right)$ and $Q_{1}\left(t\right)$ are internal circuit state components.

The matrix A is a linear constant matrix given by

(62) $$A=\left[\begin{array}{ccc} 0 & 0 & -\frac{1}{C}\\ 0 & -\frac{1}{\bar{R}C_{1}} & \frac{1}{C_{1}}\\ \frac{1}{L_{1}} & -\frac{1}{L_{1}} & -\frac{R_{1}}{L_{1}} \end{array}\right]$$

The other sources for the circuit are $s\left(t\right)$ and $b\left(Q\left(t\right)\right)$ defined as

(63) $$s\left(t\right)={\left[0,\frac{\bar{p}\left(t\right)}{\bar{R}C_{1}},0\right]}^{T}\;b\left(Q\left(t\right)\right)={\left[\frac{P\left(t\right)-\pi \left(t\right)}{\mathrm{RC}},0,0\right]}^{T}$$

The matrix A and sources $s\left(t\right)$ correspond to the circuit in Figure 3 with 2 resistors, $R_{1}\bar{R}$, and an inductor $L_{1}$ connected in series with a node between that connects to ground by way of a capacitor C. $\bar{R}$ is connected to the source $s\left(t\right)$, and $\bar{p}$ is the source voltage. The last term $Q\left(t\right)$ is the pair R and C resistor that connects to the poroelastic system and a capacitor that connects to ground. $b\left(Q\left(t\right)\right)$ is connected to the node at the negative end of the resistor $R_{1}$. That is, $R,R_{1},L_{1}$ and $\bar{R}$ are connected in series.

For this circuit system U is given by

(64) $$U=\left[\begin{array}{ccc} C & 0 & 0\\ 0 & C_{1} & 0\\ 0 & 0 & L_{1} \end{array}\right]$$

Using Equation (8) to describe the circuit ODE with the sources and inputs described in (62), (63) and initial condition $y^{0}$ our state solution is given by

(65) $$y\left(t\right)=e^{At}y^{0}+\int_{0}^{t}e^{A\left(t-\tau \right)}\left(s\left(\tau \right)+b\left(Q\left(\tau \right)\right)\right)\mathrm{d\tau}$$

### 6.1 Numerical Stability of 1D–0D Case

We have the following theorem for the unconditional stability of the operator splitting method in the 1D–0D case with (IPC) interface conditions.

Theorem 3 *The operator splitting method for the 1D–0D case with (IPC) interface conditions is unconditionally stable in the absence of external forcing with respect to the choice of the time discretization step* Δt.

Note that in 1D, the (IPC) and (PPC) interface conditions coincides, since

(66) $$P\left(t\right)=\frac{1}{\mathrm{ab}}\int_{-a/2}^{a/2}\int_{-b/2}^{b/2}p\left(c,t\right)\text{dydz}=p\left(c,t\right)$$

and interface condition Kv⋅n=p−π is equivalent to

(67) $$\mathrm{abv}\left(c,t\right)=\frac{1}{R}\left(P\left(t\right)-\pi \left(t\right)\right)=Q\left(t\right).$$

Hence, the stability of this numerical scheme follows from the stability of the numerical scheme with (PPC) interface conditions, which can be found in [18].

## 7 Numerical Experiments

This section reports numerical experiments aimed at assessing the implications of adopting PPC or IPC as interface conditions in the 3D–0D coupled problems considered. We recall that the OS approach presented in this manuscript attains unconditional stability with respect to the choice of the time step by solving Step 1 and Step 2 sequentially, without the need of sub‐iterating between them [18]. Therefore, since the computational costs of implementing the IPC/PPC interface conditions, as detailed below, and solving Step 2 are negligible with respect to solving the linear system coming from the spatial discretization of Step 1, our multiscale approach does not introduce any significant additional complexity compared to solving the Biot problem without any coupling. In the numerical treatment of the operator splitting approach, we utilize (i) a Hybridizable Discontinuous Galerkin method (HDG) for the spatial discretization of the 3D part of the coupled system; and (ii) the Backward Euler method for temporal discretization. To this end, Equation (36) is first rewritten in mixed form:

$$\begin{array}{l} AT+P_{T}pI-E=0\;\text{in}\;\Omega \times \left(t^{n},t^{n+1}\right)\\ \;\nabla \cdot T+F=0\;\text{in}\;\Omega \times \left(t^{n},t^{n+1}\right)\\ \;v+k\nabla p=S\;\text{in}\;\Omega \times \left(t^{n},t^{n+1}\right)\\ \;{\left(\nabla \cdot u\right)}_{t}+\nabla \cdot v=S\;\text{in}\;\Omega \times \left(t^{n},t^{n+1}\right)\\ \;\frac{\mathrm{d\pi}}{\mathrm{dt}}=\frac{Q\left(t\right)}{C}\;\text{in}\;\left(t^{n},t^{n+1}\right), \end{array}$$

where A is the compliance tensor, $P_{T}=\frac{1}{3\lambda +2\mu }$, and E is the symmetric part of the gradient of u. Then, the above system is discretized using a Discontinuous Galerkin approach, coded with the use of Lagrange multipliers to weakly enforce the continuity across inter‐element faces, and then hybridized so that the only global variable is the collection of Lagrange multipliers. Details about the HDG discretization and its implementation for the operator splitting approach equipped with PPC interface conditions (57) can be found in [18]. Here, we briefly describe how IPC interface conditions are implemented. The interface conditions on Σ discretized by the Backward Euler method read:

$$\begin{array}{c} u^{n+1}\left(x\right)=0,Kv^{n+1}\left(x\right)\cdot n=p^{n+1}\left(x\right)-\pi^{n+1},\\ \frac{1}{m\left(\Sigma \right)}\int_{\Sigma }p^{n+1}\left(x\right)\;dx=P^{n+1},Q^{n+1}=\frac{P^{n+1}-\pi^{n+1}}{R}. \end{array}$$

The scalar ODE to be solved in Step 1 is discretized as follows

$$\frac{\pi^{n+1}-\pi^{n}}{\Delta t}=\frac{Q^{n+1}}{C},$$

which, combined with the definition of $Q^{n+1}$, gives

$$\pi^{n+1}={\left(1+\frac{\Delta t}{\mathrm{RC}}\right)}^{-1}\left(\frac{\Delta t}{\mathrm{RC}}P^{n+1}+\pi^{n}\right).$$

By plugging the last equation into the second interface condition, and using the integral condition on $p^{n+1}$, we obtain

$$Kv^{n+1}\left(x\right)\cdot n-p^{n+1}\left(x\right)+\frac{\Delta t}{\left(\mathrm{RC}+\Delta t\right)\;m\left(\Sigma \right)}\int_{\Sigma }p^{n+1}\left(x\right)\;dx=-\frac{\mathrm{RC}}{\mathrm{RC}+\Delta t}\pi^{n},$$

which can be easily implemented in the HDG discretization. Discontinuous finite elements of degree 1 are employed in all the numerical experiments illustrated in the remainder of the section. The three‐dimensional computational domain analyzed in Section 7.1 is discretized with a fully unstructured grid made of 1577 tetrahedra whereas in the test case studied in Section 7.2 a finer unstructured grid with 97806 tetrahedra is used. In every simulation, a time step of Δt=0.02s is used. Results are expressed in SI base and derived units (unit of time = s, unit of velocity = m s^−1^, unit of pressure = Nm^−2^).

Remark 5 In another publication (see [54], sec. 3.3.4, validation test cases 1 and 3), we show stability and accuracy in space and time of an HDG method identical to the one used here, provided that the permeability tensor is equal to (a multiple of) the identity tensor. Results from [54] can be transferred to the PPC case using minor modifications of the proofs used in [55], whereas, for the IPC case, we can exploit the fact that implementing it resorts to solving the Biot problem with a Robin‐like boundary condition, as it is explained above.

### 7.1 Simulation of the 3D–0D Biot Coupled System With One–Dimensional Solution

**FIGURE 4:**
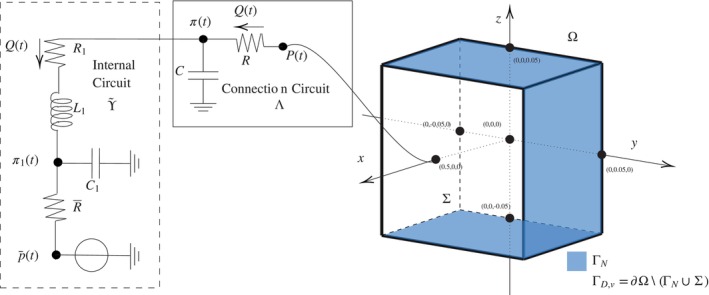
Diagram for the simplified multiscale coupling.

We begin by studying the 3D–0D problem that led to the 1D–0D example considered in Section 6. Model parameters used in the simulations are summarized in the table below (see Figure 4).

**Table:** 

Model parameters for numerical simulations			
Parameter	Description	Value	Unit
$T_{\mathrm{end}}$	Final time	10	s
k	Permeability	1	$m^{4}N^{-1}s^{-1}$
K	Aggregate modulus	1	${\mathrm{Nm}}^{-2}$
$R_{i}$	Resistance	1	${\mathrm{Nsm}}^{-5}$
$C_{i}$	Capacitance	$10^{-1}$	$m^{5}N^{-1}$
$L_{i}$	Inductance	1	${\mathrm{Ns}}^{2}m^{-5}$

**FIGURE 5:**
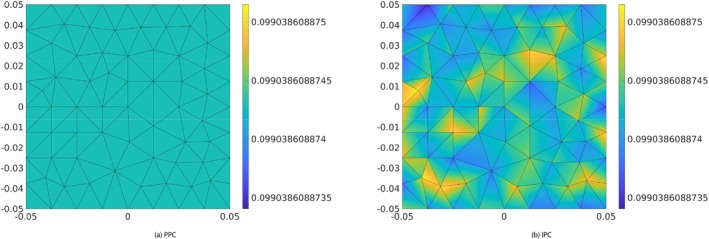
Test 7.1: Fluid pressure at the 3D–0D interface (x=0.5) at time t=10s, time step Δt=0.02s. Color maps represent the pressure magnitude (units = Nm^−2^).

**FIGURE 6:**
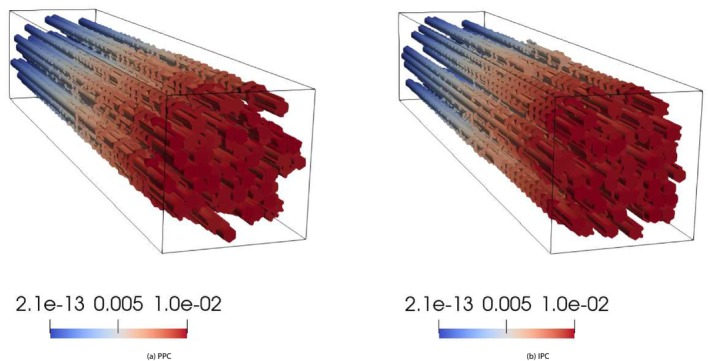
Test 7.1: Streamlines of the fluid velocity at time t=10s, time step Δt=0.02s. Color map represents magnitude of the velocity (units = ms^−1^).

**FIGURE 7:**
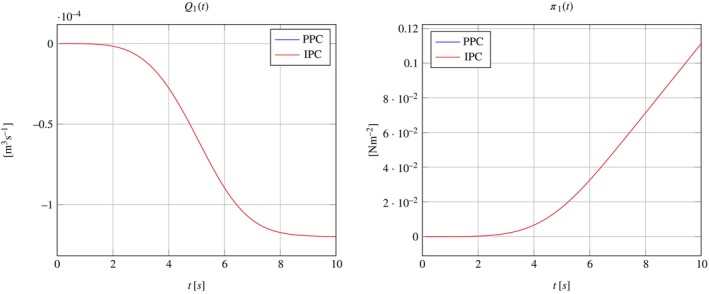
Plots of $Q_{1}\left(t\right)$ and $\pi_{1}\left(t\right)$ with the solutions obtained for PPC and IPC in the case of one‐dimensional solution.

Figure 5 shows the color maps of the fluid pressure at the 3D–0D interface obtained with PPC (a) and IPC (b) at time t=10s. The pressure distribution on the interface is constant in space when PPC are adopted, see Figure 5a, which is a confirmation that PPC work as expected. When IPC are adopted, the pressure is discontinuous by construction across all the mesh triangular faces, included those lying on the 3D–0D interface. In particular, it is taken in the following space (see [18])

$$M_{h}=\{\mu \in L^{2}\left({ℱ}_{h}\right)|\mu \in P_{1}\left(F\right)\;\forall F\in F_{h}\},$$

where ${ℱ}_{h}$ is the set of all mesh triangular faces, and $P_{1}\left(F\right)$ is the set of 2D polynomials of degree 1 defined on face F. This is the reason why the pressure does not result to be uniformly distributed over the interface, which translates into the different color shadings in Figure 5b. This is to be expected, since IPC only enforce the continuity of pressure in an integral sense. However, the pressure differences across the interface are negligible; they are less than $10^{-10}$ Pa, which is less than 0.0000001% of the overall pressure magnitude of approximately 0.1 Pa. Figure 6 further supports these findings, showing consistent streamlines of the scaled Darcy velocity throughout the 3D domain at time t=10s. Also, Figure 7 show that the results obtained for $Q_{1}\left(t\right)$ and $\pi_{1}\left(t\right)$ in the PPC and IPC cases are indistinguishable.

These results show that the numerical simulations are consistent with the analytical solution given in Section 6. When the domain geometry and the external forcing are conducive of an overall one‐dimensional solution, then adopting PPC or IPC to model the interface coupling would yield equal results up to minimal differences due to numerical approximations.

### 7.2 Simulation of the 3D–0D Biot Coupled System With a Three–Dimensional Solution

In this Section, we consider a test case in which the solution is driven by a truly 3D external forcing. The geometry of the 3D domain is the same as that considered in Section 7.1, where $\Omega =\left(0,c\right)\times \left(-a/2,a/2\right)\times \left(-b/2,,,b,/,2\right)$ with a=b=0.1 m and c=0.5 m. The 0D system is also the same. The 3D nature of the solution comes from the boundary conditions, which now are given by

$$\begin{array}{l} \mathrm{Tn}=0\;v\cdot n=0\;\mathrm{on}\;\Gamma_{N}\times \left(0,T\right),\\ \;u=0,\;v\cdot n=\psi \left(x,t\right),\;\mathrm{on}\;\Gamma_{D,v}\times \left(0,T\right), \end{array}$$

with

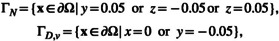

and

$$\psi \left(x,t\right)=\left(\begin{array}{c} -0.2\cos\left(10\mathrm{\pi y}\right)\cos\left(10\mathrm{\pi z}\right)\left(1+0.5\cos\left(0.2\mathrm{\pi t}\right)\right)\;\text{for}\;x=0,\\ 0.2e^{-1000\left({\left(x-0.45\right)}^{2}+z^{2}\right)}\left(1-0.5\sin\left(0.3\mathrm{\pi t}\right)\right)\;\text{for}\;y=-0.05. \end{array}\right.$$

**FIGURE 8:**
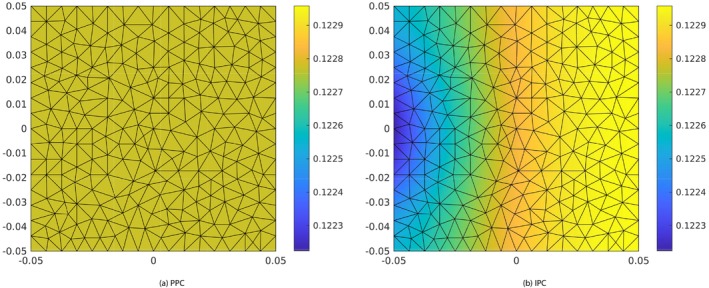
Test 7.2: Fluid pressure at the 3D–0D interface (x=0.5) at time t=10s, time step Δt=0.02s (units = Nm^−2^).

The pressure distributions obtained by adopting PPC and IPC are reported in Figure 8 for t=10s. As expected, the interface pressure is constant in the PPC case, see Figure 8a, while it exhibits variations in space in the IPC case, see Figure 8b. As a consequence, the velocity streamlines behave differently at the interface when the two types of interface conditions are used.

**FIGURE 9:**
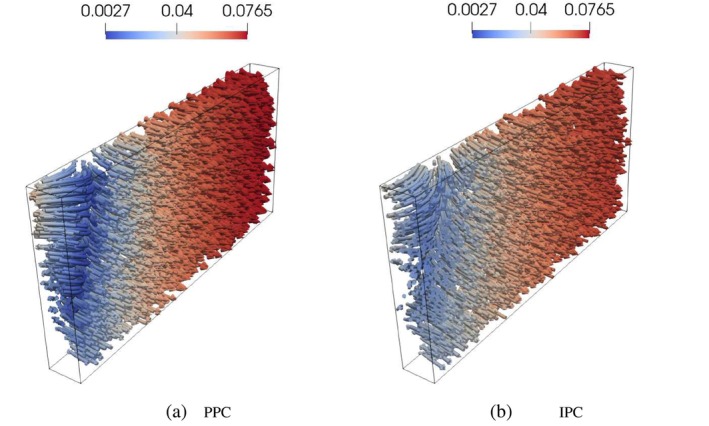
Test 7.2: Streamlines of the fluid velocity in the region x∈Ω∣0.49≤x≤0.5,z≤0 at time t=10s, time step Δt=0.02s. Color map represents magnitude of the velocity (units = ms^−1^).

**FIGURE 10:**
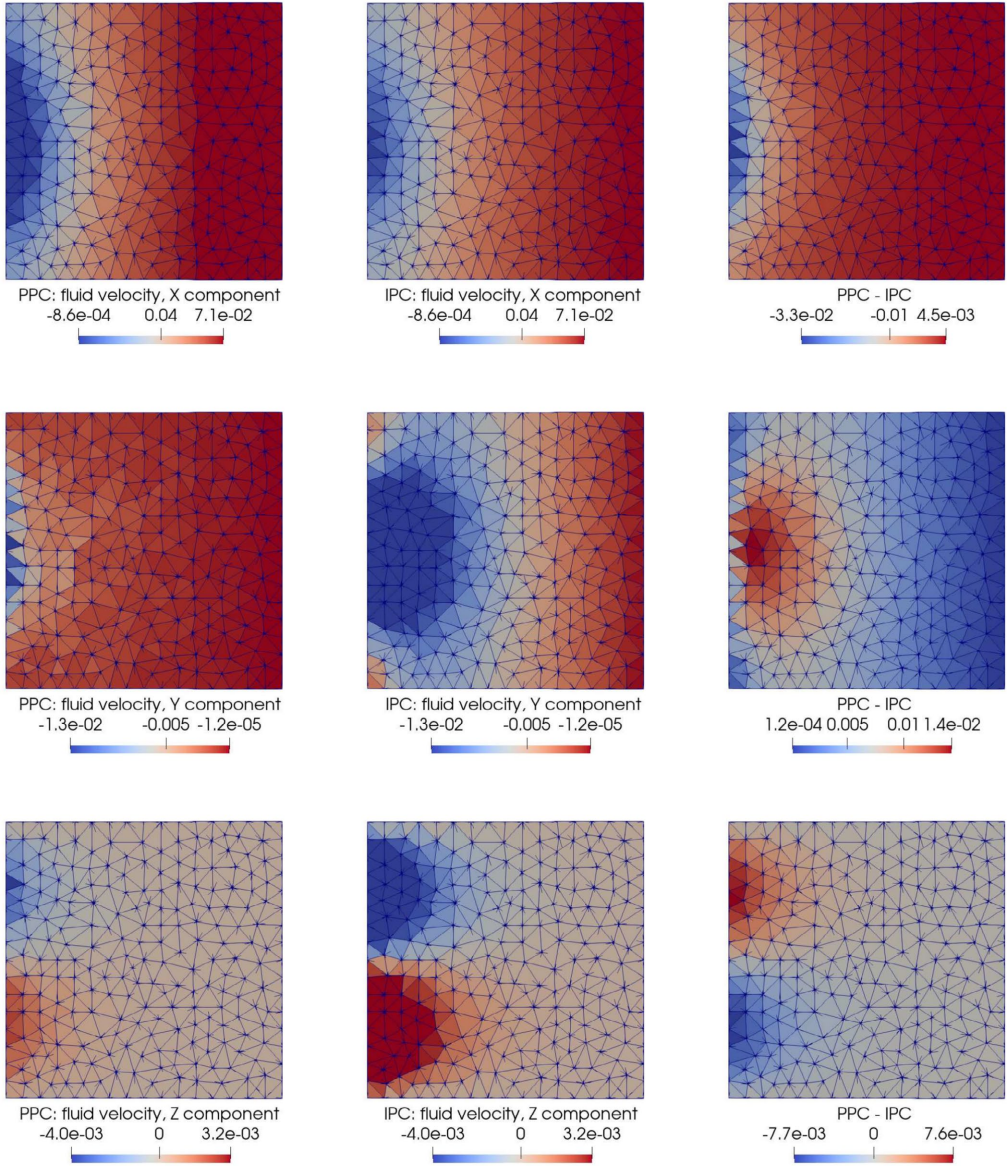
Test 7.2: Average Darcy velocity over the mesh elements sharing a face with the 3D–0D interface (x=0.5) at time t=10s, time step Δt=0.02s (units = ${\mathrm{ms}}^{-1}$). Non‐smooth color variations between neighboring triangles appear because the velocity is discontinuous by construction across mesh tetrahedra. Running simulations on a finer mesh would dump these variations, thanks to the stability and accuracy of the HDG method we used (see Remark 5).

**FIGURE 11:**
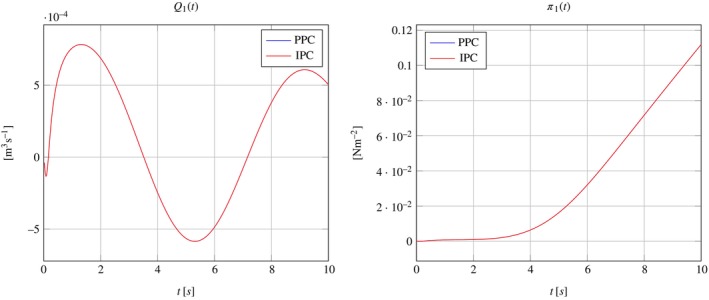
Plots of $Q_{1}\left(t\right)$ and $\pi_{1}\left(t\right)$ with the solutions obtained for PPC and IPC in the case of fully three‐dimensional solution.

The Darcy velocity at the interface is visualized as streamlines in Figure 9, with a zoomed view over a small domain slice close to the boundary, and as colormaps by components over the entire interface in Figure 10. Figure 11 show plots of $Q_{1}\left(t\right)$ and $\pi_{1}\left(t\right)$ with the solutions obtained for PPC and IPC superimposed. In the PPC case, the constraint for the pressure to be constant on the interface drives the velocity field to adjust and accommodate the continuity of mass within such constraint. Conversely, in the IPC case, the velocity field will ensure the continuity of mass, while the pressure will redistribute over the interface to guarantee the continuity of pressure.

## 8 Conclusions

This work shows that different 3D–0D interface conditions give rise to different solutions, especially when the problem to be solved does not exhibit any particular geometrical and/or physical symmetry. Consequently, the choice of which interface conditions to adopt, whether PPC or IPC, should be considered part of the modeling process. The different interface coupling conditions are introduced at the continuous level in Section 2. The properties of the physical energy of the coupled system are also elaborated at the continuous level in Section 3. The specific time and space discretizations adopted to solve the coupled problem, presented in Section 5, are meant to prevent disruption of such physical properties at the discrete level and do not limit the applicability of the simulation findings.

In the context of tissue perfusion, the interface represents portion of the tissue where fluid moves between larger vessels, modeled as a 0D circuit, and smaller capillaries, modeled as pores within the 3D domain. Within the eye, the tissues are subject to mechanical deformations due to the action of external pressures acting on them, such as the intraocular pressure and the cerebrospinal fluid pressure. Such deformations may affect the pore, or capillary, size to a different extent across the tissue, including the 3D–0D interface, thereby leading to changes in the pressure distribution. Thus, the choice of IPC to model the 3D–0D interface seems more appropriate when modeling tissue perfusion in the eye.

It is worth noting that the extension of the model to different types of tissues, which is important for the analysis of many pathological conditions, is facilitated by the operator splitting approach adopted in the manuscript. Specifically, Step 1 isolates the solution of the 3D model for the tissue; consequently, any changes in the constitutive modeling for the tissue will only impact Step 1 of the overall algorithm. This is very important, for example, when studying tumor growth [56], heart perfusion [13], or tissue deformation during subcutaneous injection [57].

It is important to realize, though, that with more realistic tissue models also come additional computational challenges. Strategies to counter these challenges are active area of research, for example, [58]. The implementation of such models would greatly benefit from software exploiting parallel multiprocessing, hybrid Message‐Passing‐Interface (MPI) and multi‐threading computing, hybrid CPU‐GPU computing. A related key aspect is to choose linear/nonlinear solvers particularly suited for a parallel setting, for example, iterative methods accelerated by domain‐decomposition [59] or algebraic multigrid preconditioners [60]. Additionally, since realistic and case‐specific simulations might require to repeat calculations for many different values of model parameters, it might be convenient to resort to surrogate strategies like reduced basis methods [61], uncertainty quantification [62], and machine learning [63], which require computationally intense preprocessing steps to be done once and for all, but then allow fast and cheap model evaluations at the online stage.

## Ethics Statement

The authors have nothing to report.

## Conflicts of Interest

The authors declare no conflicts of interest.

## Data Availability

The data that support the findings of this study are openly available in hdg3d_poroviscoelasticity at https://bitbucket.org/dadda/hdg3d_poroviscoelasticity/src/master/.
